# Delivery of functional exogenous proteins by plant-derived vesicles to human cells in vitro

**DOI:** 10.1038/s41598-021-85833-y

**Published:** 2021-03-22

**Authors:** Luiza Garaeva, Roman Kamyshinsky, Yury Kil, Elena Varfolomeeva, Nikolai Verlov, Elena Komarova, Yuri Garmay, Sergey Landa, Vladimir Burdakov, Alexander Myasnikov, Ilya A. Vinnikov, Boris Margulis, Irina Guzhova, Alexander Kagansky, Andrey L. Konevega, Tatiana Shtam

**Affiliations:** 1 Petersburg Nuclear Physics Institute named by B.P. Konstantinov of National Research Centre “Kurchatov Institute”, mkr. Orlova roscha 1, 188300 Gatchina, Russian Federation; 2 Institute of Cytology of Russian Academy of Sciences, Tikhoretsky Ave. 4, 194064 St. Petersburg, Russian Federation; 3 National Research Center “Kurchatov Institute”, Akademika Kurchatova pl. 1, 123182 Moscow, Russian Federation; 4 Center for Genomic and Regenerative Medicine, School of Biomedicine, Far Eastern Federal University, Vladivostok, Russian Federation; 5 Laboratory of Molecular Neurobiology, School of Life Sciences and Biotechnology, Shanghai Jiao Tong University, Shanghai, China; 6 Shubnikov Institute of Crystallography, Federal Scientific Research Centre ‘Crystallography and Photonics’, Russian Academy of Sciences, Leninskiy prospect, 59, 119333 Moscow, Russian Federation; 7 Moscow Institute of Physics and Technology, Institutsky lane 9, Dolgoprudny, 141700 Moscow, Russian Federation; 8 Peter the Great St. Petersburg Polytechnic University, Politehnicheskaya 29, St. Petersburg, Russian Federation; 9 Kurchatov Genome Center-PNPI, 188300 Gatchina, Russian Federation

**Keywords:** Biological techniques, Cell biology, Plant sciences, Molecular medicine, Nanoscience and technology

## Abstract

Plant-derived extracellular vesicles (EVs) gain more and more attention as promising carriers of exogenous bioactive molecules to the human cells. Derived from various edible sources, these EVs are remarkably biocompatible, biodegradable and highly abundant from plants. In this work, EVs from grapefruit juice were isolated by differential centrifugation followed by characterization of their size, quantity and morphology by nanoparticle tracking analysis, dynamic light scattering, atomic force microscopy and cryo-electron microscopy (Cryo-EM). In Cryo-EM experiments, we visualized grapefruit EVs with the average size of 41 ± 13 nm, confirmed their round-shaped morphology and estimated the thickness of their lipid bilayer as 5.3 ± 0.8 nm. Further, using cell culture models, we have successfully demonstrated that native grapefruit-derived extracellular vesicles (GF-EVs) are highly efficient carriers for the delivery of the exogenous Alexa Fluor 647 labeled bovine serum albumin (BSA) and heat shock protein 70 (HSP70) into both human peripheral blood mononuclear cells and colon cancer cells. Interestingly, loading to plant EVs significantly ameliorated the uptake of exogenous proteins by human cells compared to the same proteins without EVs. Most importantly, we have confirmed the functional activity of human recombinant HSP70 in the colon cancer cell culture upon delivery by GF-EVs. Analysis of the biodistribution of GF-EVs loaded with ^125^I-labeled BSA in mice demonstrated a significant uptake of the grapefruit-derived extracellular vesicles by the majority of organs. The results of our study indicate that native plant EVs might be safe and effective carriers of exogenous proteins into human cells.

## Introduction

Extracellular vesicles (EVs), including exosomes, are nanoscale membrane-enclosed particles implicated in intercellular communication to facilitate transport of proteins and genetic material^1–3^. Due to their natural properties, exosomes are capable of migrating from one cell to another, carrying their contents across the cell membrane, and delivering of biologically active cargoes^4^. Since exosomes offer distinct advantages for the efficient and safe delivery of biomolecules, the interest in using them as carriers has exploded in recent years^5,6^. Mammalian EVs have been reported to be used for the delivery of siRNAs^7^, miRNAs^8^, proteins^9^, small molecule drugs^10^, and CRISPR/Cas9 molecular toolkits^11^ with the ultimate goal to develop novel therapeutics (reviewed in ^12^).

However, there is a number of limitations associated with the use of human exosomes as drug delivery vehicles. One of the major challenges in developing exosome-based formulations is the concern whether and how the sufficient amounts of human exosomes can be generated in vitro or isolated from biological fluids. Indeed, the exosome yield per unit of starting material will impact the final production cost as well as clinical applications. In this respect, the choice of alternative sources of nanovesicles is crucial. The use of vesicles innately formed in plant cells as delivery agents could potentially solve problems associated with the existing nano-delivery systems. Firstly, the natural origin of plant-derived extracellular vesicles (PEVs) allows their isolation from affordable edible plants in significant quantities and eliminates any possible concerns regarding their toxicity^13–15^. Secondly, plant vesicles are natural carriers of different biomolecules, including small non-coding RNAs^16^ and, therefore, are designed to maintain stability of their molecular cargo in facilitating cell-to-cell communication^17,18^. These properties of PEVs suggest the possibility of their applications as carriers for ectopic cargo delivery. In general, PEVs are much less studied than the vesicles secreted by mammalian cells, and their structural and functional features remain to be elucidated in detail.

Due to a possible aggregation during purification and a relatively low exogenous cargo loading capacity, some doubts had been raised about using native plant vesicles as drug carriers. Indeed, under the necessary for isolation ultracentrifugation conditions, these PEVs may aggregate thus hindering their use for intravenous administration. These problems have been resolved by introducing additional purification steps while isolating PEVs^19,20^ or by reassembling plant nanovectors from the molecules that make up their membrane^13^. The nanovectors constructed from grapefruit particle-derived lipids were demonstrated as delivery system for chemotherapeutic drugs and siRNAs^13^.

In the present study, we evaluated the potential of native, chemically unmodified plant vesicles as a delivery system for exogenous proteins in vitro and in vivo. We have isolated extracellular vesicles from grapefruit juice by differential centrifugation and characterized them in size, quantity, and morphology by nanoparticle tracking analysis (NTA), dynamic light scattering (DLS), atomic force microscopy (AFM), and cryo-electron microscopy (Cryo-EM). Finally, native grapefruit-derived extracellular vesicles (GF-EVs) have been investigated for their potential use in delivery of functional proteins into the human cells in vitro. Using tissue culture models, we have shown that GF-EVs are highly efficient in the delivery of exogenous recombinant human heat shock protein 70 (HSP70) into various human cells. Additionally, the biodistribution of GF-EVs loaded with exogenous protein in mice has been evaluated.

## Results

### Characterization of grapefruit-derived nanovesicles

**Figure 1:**
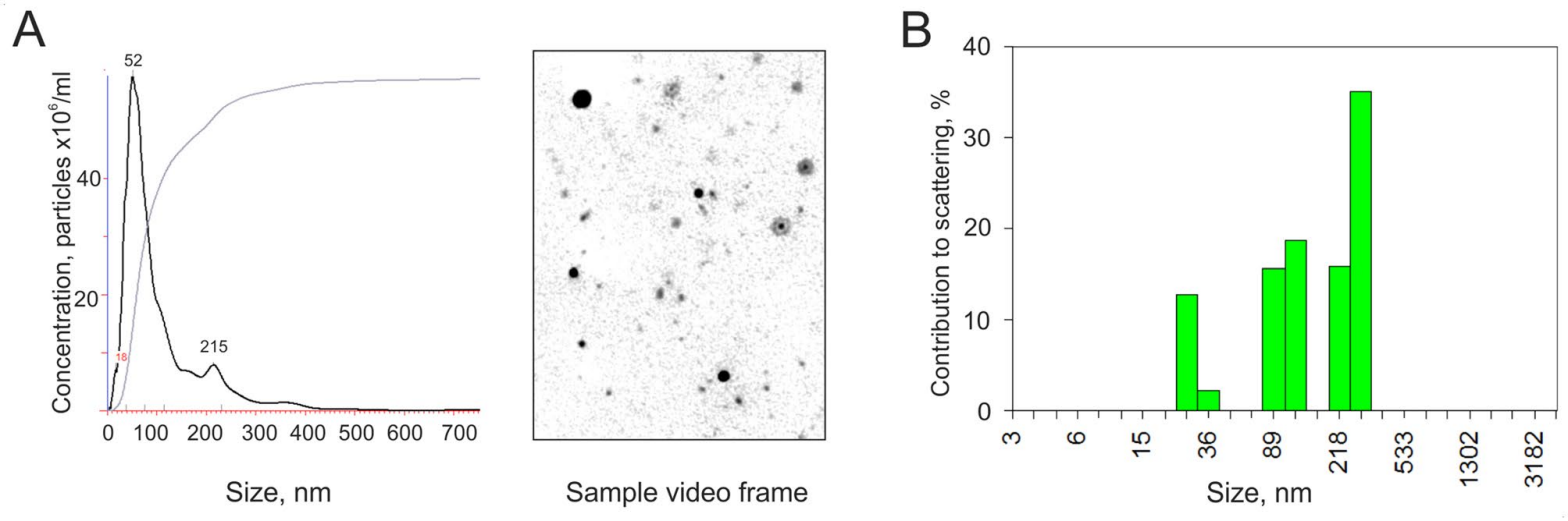
Characterization of grapefruit-derived particle size and concentration. (**A)** Nanoparticle tracking analysis (NTA) of size and concentration in the sample of isolated GF-EVs. (**B)** A typical example of the size distribution of isolated extracellular vesicles from grapefruit juice measured by dynamic light scattering (DLS). The majority of particles (90 ± 4% contribution by mass) had a size of about 30 nm.

Vesicles were isolated from 400 mL of grapefruit juice by sequential ultracentrifugation according to the purification protocol for edible plant-derived exosome-like nanoparticles described earlier^16^ with some modifications. In order to characterize grapefruit-derived extracellular vesicles, their size distribution and concentration were measured by NTA. In the purified samples of GF-EVs the following parameters were measured: size mode and concentration of vesicles in suspension. These parameters were determined as 52 ± 8 nm and 5.7 ± 0.7 × 10^13^ particles/mL, respectively (Fig. 1A). Since the NTA method gives a wide size distribution, we further performed the sizing of vesicles isolated from grapefruit using DLS. Three distinct peaks were detected by DLS: 28 ± 10 nm, 80 ± 6 nm, and 270 ± 46 nm (Fig. 1B). The majority of particles (90 ± 4% contribution by mass) had a size of about 30 nm.

**Figure 2:**
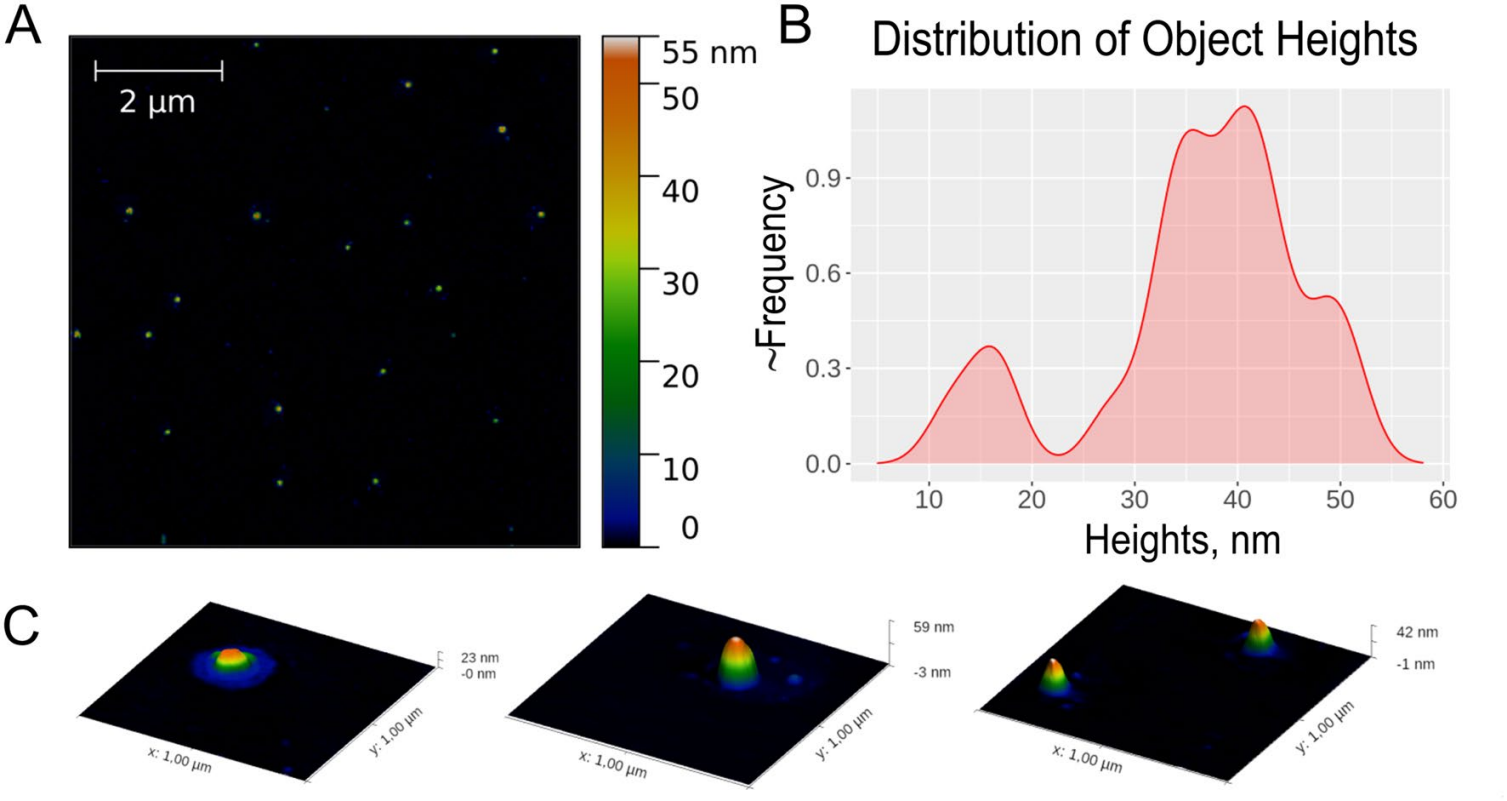
Characterization of grapefruit-derived nanoparticle size and morphology by Atomic Force Microscopy (AFM). (**A**) AFM images of grapefruit nanovesicles. (**B**) Height distribution of GF-EVs. (**C)** 3D images of some representative individual vesicles.

Surface topology of grapefruit nanovesicles was estimated by AFM. In several samples of vesicles isolated from grapefruit, we have observed individual particles of spherical shape that corresponded to vesicular topology with diameters ranging from 50 to 120 nm, and heights from 30 to 60 nm. In addition, a number of small particles with heights of about 15 nm were also observed (Fig. 2).

### Morphological characterization of GF-EVs by Cryo-EM

**Figure 3:**
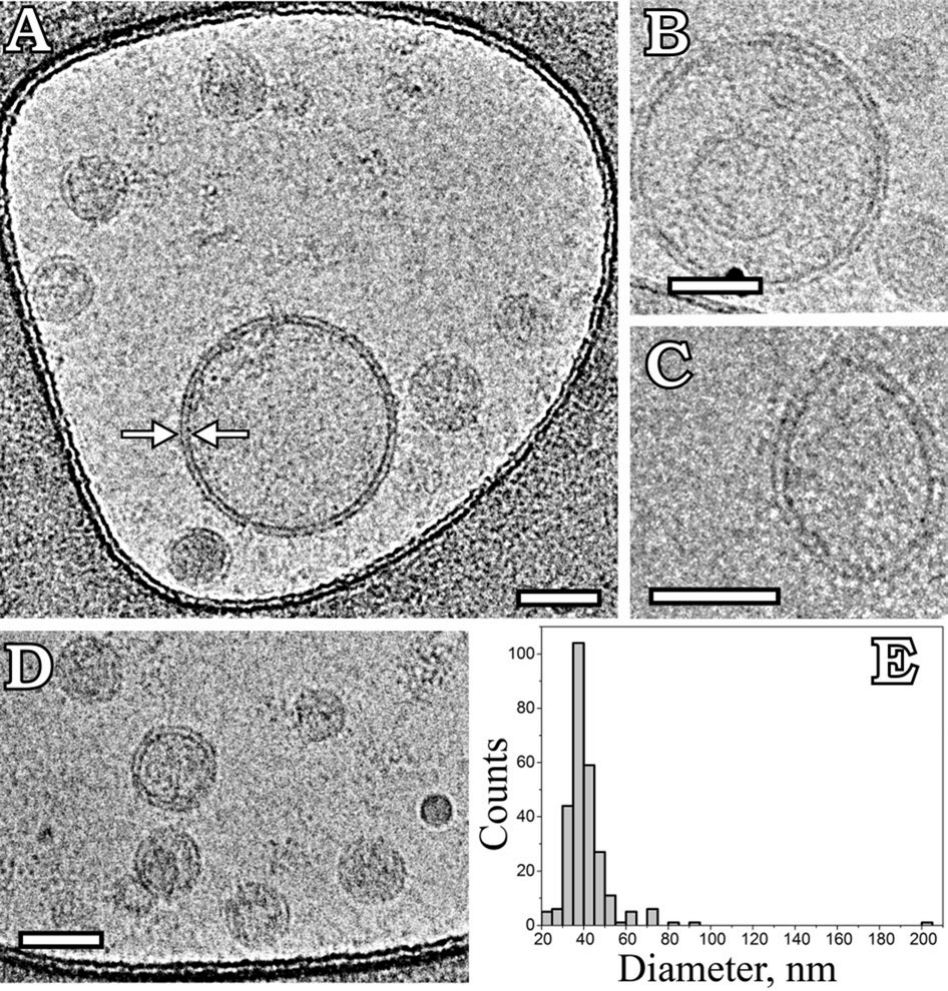
Cryo-EM images of grapefruit-derived extracellular vesicles (GF-EVs). (**A**,**B**,**D**) Round single vesicles; (**B**) double vesicle; (**C**) elliptical vesicle; (**E**) size distribution histogram. A total of 270 particles was analyzed. The arrows depict a lipid bilayer membrane of vesicle. Scale bars are 50 nm.

The morphology of grapefruit-derived particles was characterized using Cryo-EM (Fig. 3). Since the majority of the particles had a round-shaped vesicular morphology formed by a characteristic lipid bilayer with an average thickness of 5.3 ± 0.8 nm (Fig. 3A,B,D), these particles were defined as grapefruit-derived extracellular vesicles. Among them single vesicles containing electron dense material were visualized (Fig. 3A,D). The average size of GF-EVs was 41 ± 13 nm (Fig. 3E). A small number of larger vesicles (up to 200 nm) were also observed (Fig. 3A,B,E). The presence of vesicles with altered morphology, such as double (Fig. 3B) and elliptical vesicles (Fig. 3C), was insignificant.

### GF-EV-mediated delivery of exogenous proteins into human cells

**Figure 4:**
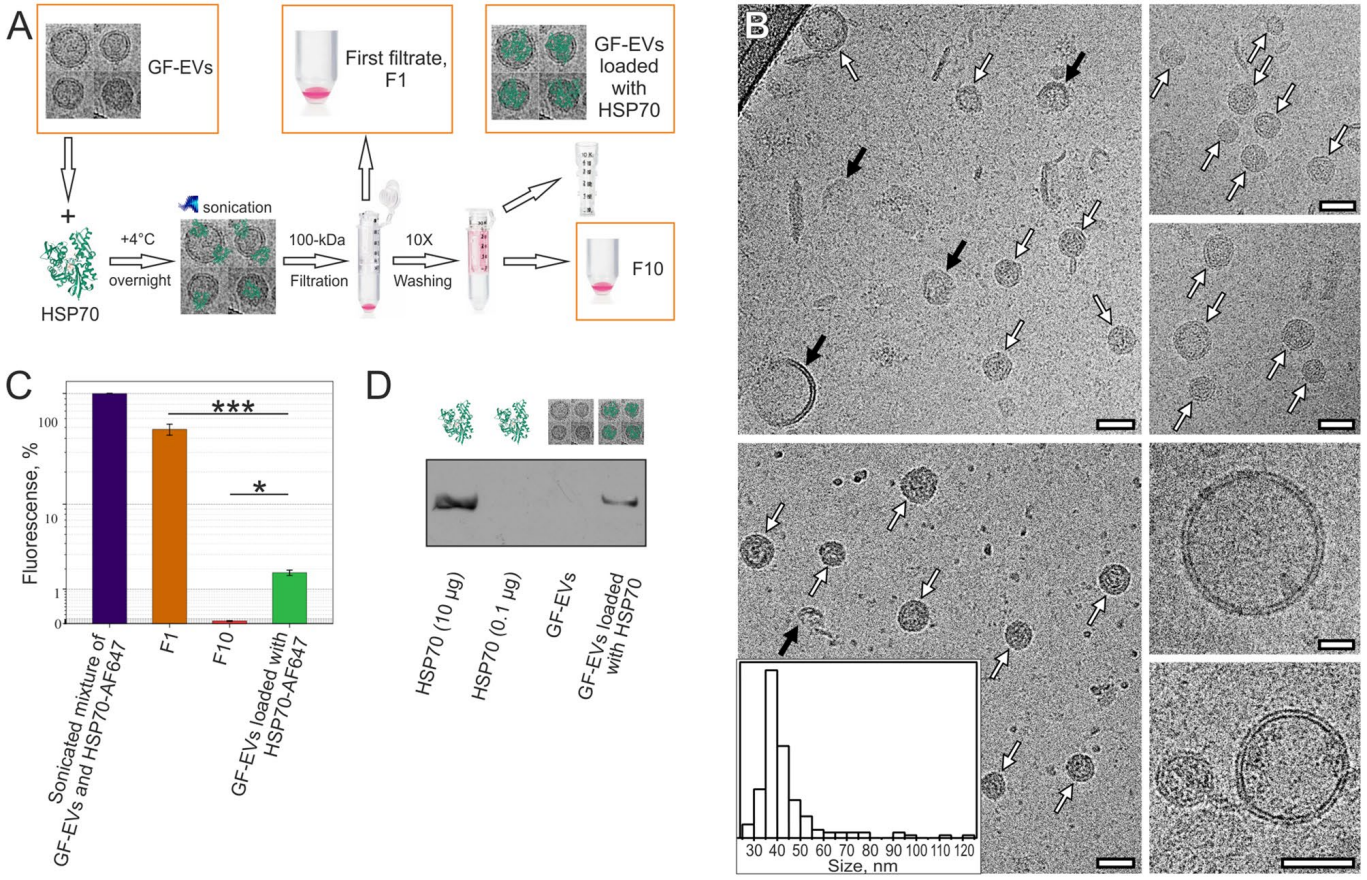
Loading efficiency of GF-EVs with HSP70 protein. (**A**) Flowchart summarizing the procedure of GF-EV loading with HSP70. (**B**) Cryo-EM images of GF-EVs loaded with HSP70. White arrows indicate vesicles with intact membrane, black arrows indicate vesicles with broken membrane. Scale bars are 50 nm. Inset—size distribution histogram. A total of 157 particles was analyzed. (**C**) Fluorescence of Alexa Flour 647 labeled HSP70 in the initial mixture and loaded samples of GF-EVs, as well as washing filtrates F1 and F10. N = 3. Data are presented as means ± standard deviation (SD). *, *p* < 0.05; ***, *p* < 0.001 as estimated by one-way ANOVA followed by multiple comparisons Tukey’s post-hoc analysis. (**D**) Western blot of HSP70 in the initial and loaded samples of GF-EVs. The full-length blot image used to generate panel is shown in Supplementary Fig. S2.

First, we investigated the possibility of loading GF-EVs with exogenous cargoes using sonication. As a procedure for transfection of the plant vesicles is not well-characterized, both Alexa Fluor 647 labeled and intact variants of HSP70 were used for empirical optimization of the protocol. The best result was demonstrated using passive protein penetration in combination with sonication. The sonicated mixture containing GF-EVs at concentration of 4.1 ± 1.4 × 10^11^ particles/mL and HSP70-AF647 at concentration of 0.1 mg/mL was purified 10 times by washing and ultrafiltration through a 100-kDa filter to eliminate the excess of free proteins. At the end of the loading procedure, each sample, including the final suspension of loaded vesicles, the first and the tenth filtrates, were adjusted to volume equal to the starting volume of the initial suspension of GF-EVs. A schematic representation of the GF-EV loading procedure with an exogenous HSP70 is shown in Fig. 4A. Sonication of vesicles, which is a part of the loading procedure, may damage the integrity of the loaded particles. Nevertheless, Cryo-EM data of GF-EVs loaded with HSP70 revealed that the fraction of broken vesicles was insignificant and the particle size (43 ± 15 nm) did not change significantly after the loading procedure (Fig. 4B). Also, the invariability of the size distribution of the loaded vesicles was confirmed by NTA (Supplementary Fig. S1). During the loading, the concentration of GF-EVs decreased from 4.1 ± 1.4 × 10^11^ particles/mL to 1.2 ± 0.2 × 10^11^ particles/mL, which was mainly due to the washing by ultrafiltration (Supplementary Fig. S1). Notably, according to the fluorometric analysis of HSP70-AF647, the first filtrate (F1) contained a significant amount of free HSP70-AF647 (about 50% of the initial amount), while the last filtrate (F10) was actually protein free (about 0.05% of the initial amount), confirming that the purification procedure used was sufficient to remove free protein (not loaded to vesicles) from the samples. At the same time, the proportion of the labeled protein loaded to the GF-EVs was about 2% of the initial amount added to the vesicle suspension before sonication (Fig. 4C). The loading efficiency of GF-EVs with intact human HSP70 protein was estimated by Western blotting using the equivalent number of particles (Fig. 4D).

**Figure 5:**
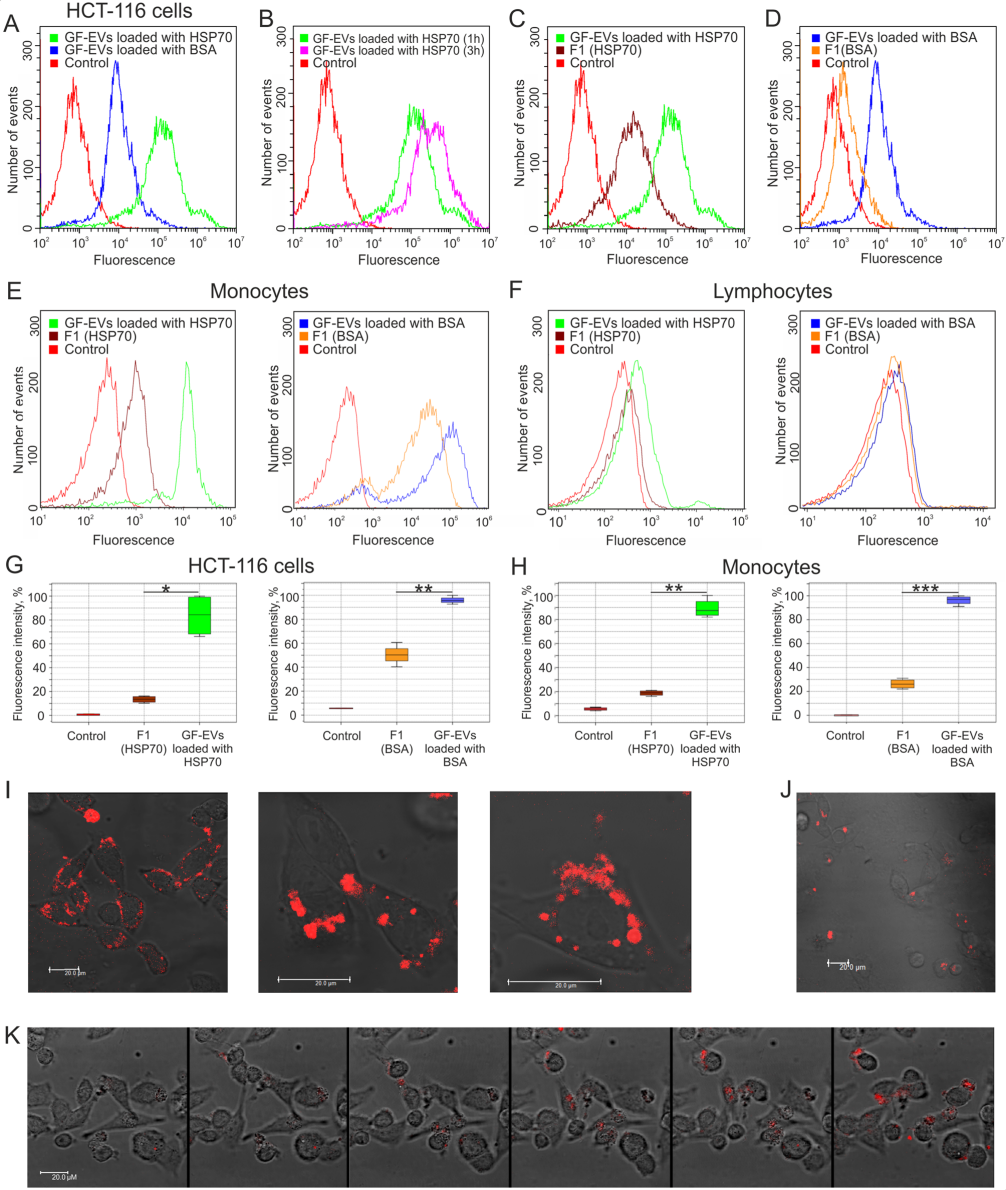
GF-EV-mediated delivery of exogenous proteins into the human cells analyzed by flow cytometry and confocal microscopy. (**A**) Incubation of colon cancer HCT-116 cells with GF-EVs loaded with BSA-AF647 or HSP70-AF647 proteins for 1 h. (**B**) Incubation of HCT-116 cells with HSP70-AF647-loaded GF-EVs for 1 and 3 h. (**C**,**D**) Incubation of HCT-116 cells with loaded GF-EVs in comparison with the free HSP70-AF647 or BSA-AF647 proteins for 1 h. (**E**,**F**) Incubation of peripheral blood mononuclear cells (**E**, monocytes and **F**, lymphocytes) with loaded GF-EVs in comparison with the free HSP70-AF647 or BSA-AF647 proteins for 1 h. (**G**,**H**) The relative intensity of fluorescence of HCT-116 cells and monocytes incubated with loaded GF-EVs in comparison with the free HSP70-AF647 or BSA-AF647 proteins. N = 2. Data are presented as means ± SD. *, *p* < 0.05, **; *p* < 0.01; ***, *p* < 0.001 as estimated by one-way ANOVA followed by multiple comparisons Tukey’s post-hoc analysis. (**I,J**) Micrographs of DLD1 cells co-cultivated with GF-EVs loaded with HSP70-AF647 (**I**) or BSA-AF647 (**J**) proteins. Scale bars are 20 µm. (**K**) Accumulation of fluorescent protein delivered into the recipient DLD1 cells by GF-EVs. Video recording frames were captured every 30 min after adding of loaded GF-EVs to the cells.

Next, GF-EVs loaded with HSP70-AF647 were co-cultured with the recipient human colon cancer HCT116 or DLD1 cells or human peripheral blood mononuclear cells (PBMC). Additionally, the uptake of BSA-AF647-loaded vesicles by human colon cancer cells was analyzed in parallel experiments. The high delivery efficiency of either protein to recipient cells by GF-EVs was confirmed by flow cytometry and confocal microscopy for all studied cell types (Fig. 5A–E, I–K), with the exception of lymphocytes (Fig. 5F). Interestingly, fluorescence signal accumulation was observed with increasing incubation time of recipient cells and HSP70-AF647-loaded GF-EVs (Fig. 5B). The results of the flow cytometry experiment showed that the fluorescence signal from labeled proteins could be detected in the cells co-cultured with the first filtrates containing the free proteins (Fig. 5C,D,E). The efficiency of cellular uptake of free HSP70 was significantly higher than free BSA. It should be noted, that in all experiments the cells were co-cultured with an equal volume of mixtures containing either protein-loaded GF-EVs or free protein. According to fluorimetry data, the amount of protein in a series of samples F1, GF-EVs loaded with protein, F10 decreased by an order of magnitude (Fig. 4C). These results reveal that the uptake of both BSA- and HSP70-loaded to GF-EVs by human cells was significantly more efficient than the uptake of free proteins (Fig. 5G,H).

A layer-by-layer scanning of colon cancer cells using confocal microscopy visualized labeled proteins within the cytoplasm of recipient cells (Fig. 5I,J). The fluorescence signal in the cells has been registered in 30 min after the start of incubation of cells together with the loaded grapefruit vesicles, and the fluorescence intensity kept increasing upon further incubation. The accumulation of fluorescent proteins delivered to the recipient cells by GF-EVs was detected in real time using confocal microscopy (Fig. 5K, Supplementary Video S1). Thus, our results indicate a highly efficient protein delivery into human cells by GF-EVs.

### Functionality of HSP70 protein delivered by GF-EVs to human colon cancer recipient cells

**Figure 6:**
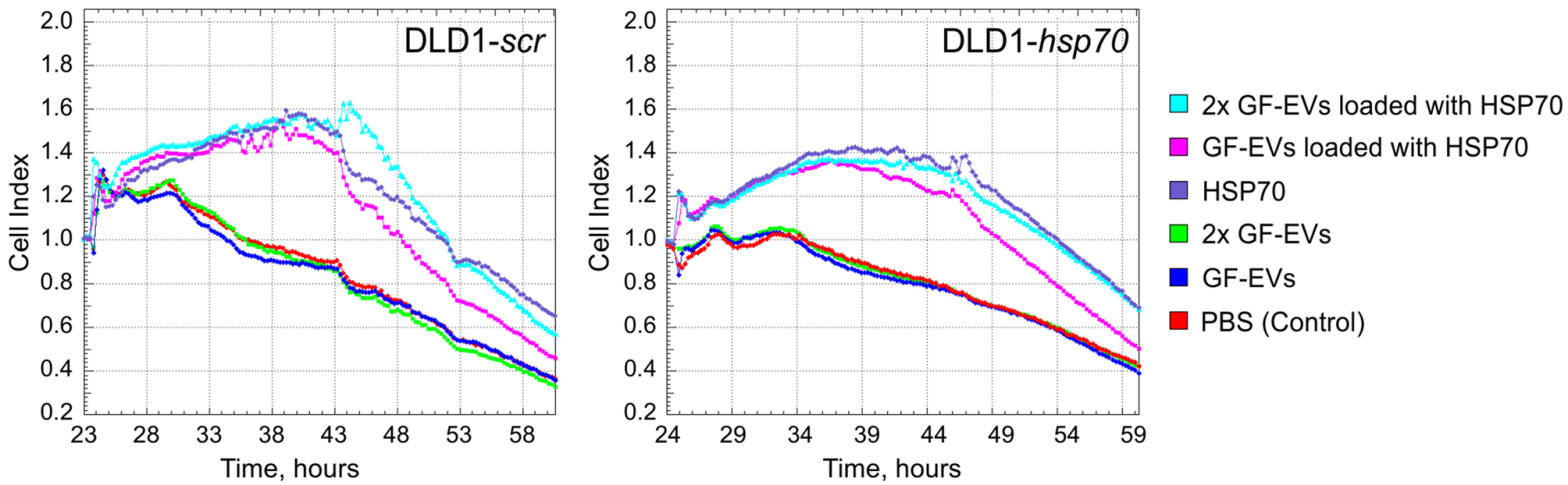
Protective effect of GF-EVs loaded with HSP70 from the etoposide-induced cytotoxicity. DLD1-*scr* (left) or DLD1-*hsp70* (right) cells were preincubated in E-plates for 19–20 h before the addition of HSP70 at concentration of 2 μg/mL or GF-EVs loaded with HSP70 (10^6^ or 2 × 10^6^ particles per cell, appr. 1 μg/mL or 2 μg/mL of HSP70 respectively); 4 h later the etoposide (EP) was added to a final concentration of 20 μM in each well. Recording with the aid of xCELLigence equipment was started immediately after drug added and lasted for 60 h. In every experiment, each point in the plot represents an average recording of two wells. In each panel, data from one of two independent experiments are presented.

In order to test whether the cargo protein HSP70 leaves the plant vesicles in the biologically functional state inside the recipient cells, we used the etoposide sensitivity in vitro model. Etoposide is a chemotherapeutic agent that inhibits cell growth and induce apoptosis in a variety of tumor cells. The HSP70 chaperone prevents etoposide-mediated anti-proliferative effect^21^. To test the functionality of the HSP70 protein delivered into the cells by GF-EVs, we analyzed the proliferative activity of colon cancer DLD1-*scr* or DLD1-*hsp70* cells in the presence of etoposide in real time with the aid of xCELLigence technology (Fig. 6). The addition of etoposide alone led to reduction of proliferation and cell death of both lines (Fig. 6, red line). The growth curves of both cell lines in the presence of etoposide and GF-EVs contained no load did not differ from the control curves, which indicates the absence of any toxic or stimulatory effect of the plant particles on the cell growth (Fig. 6, green and blue lines). However, preincubation of the cells with either free HSP70 (Fig. 6, violet lines) or different concentrations of HSP70-loaded GF-EVs (Fig. 6, magenta and cyan lines) protected the cells from the etoposide-induced cytotoxicity. Moreover, the observed protective effect was dose-dependent, correlating with the amounts of GF-EVs loaded with HSP70 (Fig. 6, magenta and cyan lines). It should be noted that the DLD1*-hsp70* cells with the depletion of endogenous HSP70 by the stable knockdown showed slightly increased sensitivity to etoposide and less proliferative activity stimulated by the exogenous HSP70 protein after the etoposide treatment compared to DLD1-*scr* cells. These experiments demonstrate that the HSP70 protein remains functional when delivered into human recipient cells by GF-EVs.

### Biodistribution of GF-EVs loaded with exogenous protein

**Figure 7:**
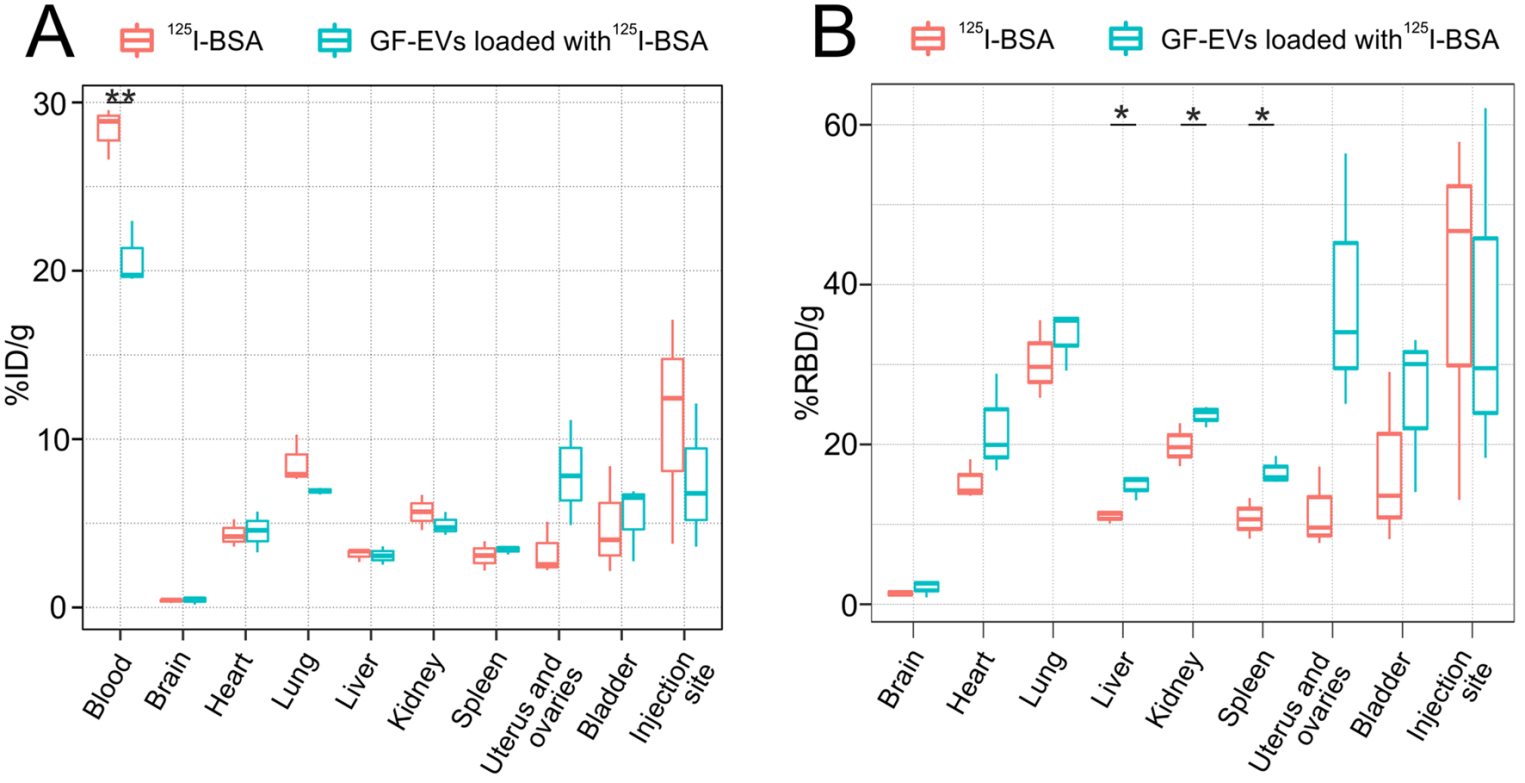
Biodistribution of GF-EVs loaded with ^125^I-labeled BSA and free ^125^I-BSA in mice 2 h after intravenous administration. Analysis of the radioactive protein abundance in different tissues expressed as a percentage of injected dose (ID) per gram of tissue (**A**) or as a percentage of relative blood dose (RBD) per gram of tissue (**B**). N = 3. Data are presented as means ± SD. *, *p* < 0.05; **, *p* < 0.01 as estimated by unpaired Student’s t-test analysis.

Next, we investigated the tissue distribution of GF-EVs loaded with a radiolabeled exogenous protein. Bovine serum albumin was labeled with radioactive ^125^I, purified from free iodine by gel filtration chromatography, loaded to GF-EVs and injected intravenously into mice. Iodine-labeled free protein was used in a parallel experiment for comparative analysis. Biodistribution of GF-EVs loaded with ^125^I-labeled BSA (^125^I-BSA) was assessed quantitatively using ex vivo gamma counting (Fig. 7A). Two hours after injection, a high percentage (> 5% of total dose) of radioactivity was observed in the lung, bladder, uterus and ovaries. A considerable amount of radioactivity was also observed in the liver, spleen, kidneys, and heart. A small portion of the vesicles loaded with ^125^I-BSA was detected in the brain samples.

Interestingly, when normalized to the initial dose of the radioactive protein, the concentration of ^125^I-BSA-loaded GF-EVs was significantly lower compared to free ^125^I-BSA in the blood, and had a tendency towards decrease in the injection site (Fig. 7A). These observations suggested that GF-EVs leave the bloodstream and the injection site more efficiently than free protein. However, when the data were normalized on the blood radioactive protein content and presented as a ratio of the dose detected in gram of tissue and the dose recorded in gram of blood (Fig. 7B), we could detect that already 2 h after injection, GF-EVs loaded with ^125^I-BSA accumulated significantly more efficiently than free ^125^I-BSA in liver, kidney and spleen. Accordingly, tendencies towards more effective uptake of GF-EVs loaded with ^125^I-BSA were also apparent for other organs (Fig. 7B). In conclusion, vesicles isolated from grapefruit juice by ultracentrifugation, loaded with exogenous protein and injected intravenously into an animal, efficiently penetrate into various tissues.

## Discussion

Over the last decade, extracellular vesicles, especially human exosomes, were considered promising carriers of different exogenous bioactive molecules to the human cells. Among these agents, therapeutic molecules of RNA, peptides or proteins are most problematic for delivery by standard methods. Human EVs from various biofluids are well studied^2^, and much progress has been achieved in using them as drug delivery systems^22,23^. In recent years, different strategies of exosome loading with various exogenous cargoes have been developed and optimized^12,24^. Haney et al.^9^ reported the high efficiency of sonication, extrusion, or permeabilization with saponin for loading human macrophage-derived exosomes with catalase. Furthermore, intranasal administration of catalase-loaded exosomes led to behavioral recovery in a murine model of Parkinson’s disease demonstrating that exosome can cross the blood brain barrier and might be used to treat neurological disorders^9^.

However, in order to expand therapeutic applications, further development of the EV-based delivery techniques is required, especially towards finding new ways to obtain large amounts of pure exosomes. Hence, in the view of their efficiency and abundance, EVs isolated from edible plants are particularly promising for these purposes^25^. Indeed, plant-derived exosome-like nanoparticles are also biocompatible and biodegradable, as are exosomes of animal origin, while having several distinct advantages. PEVs can be isolated in large amounts, thus making plants the raw materials of choice to isolate safe and effective vehicles for the delivery of therapeutic agents.

In recent years, many research groups investigated the molecular content of PEVs from various plant sources, mostly edible, such as fruits and vegetables, starchy roots and tubers, nuts and seeds, fresh and dried plants. Some studies demonstrated that PEVs release is stimulated by pathogen infection and stress, while others—that plant EVs contain proteins, various RNAs, polysaccharides, and lipid signals, related to their roles in plant defense^26,27^. Analysis of protein contents of PEVs isolated from different sources revealed several interesting proteins, which are potentially considered as specific markers, such as PENETRATION1, Patellins 1–3, Clathrin heavy chain, as well as heat shock proteins^28,29^. Although the lipid composition of plant vesicles is different from that of mammalian exosomes, the efficiency of their internalization by animal cells has been confirmed in experiments with PKH26-labeled particles and with delivery of chemotherapy agent methotrexate^20^. Grapefruit-derived nanovesicles have been studied previously in several works^13,20,30^. Using both in vitro and in vivo models, Wang and coauthors have shown that nanoparticles reconstructed from grapefruit-derived lipids are highly efficient for delivering a variety of therapeutic agents, including drugs, DNA expression vectors, siRNA, and antibodies^13^. In another work^30^, the same research group demonstrated a tumor-targeted delivery of grapefruit-derived nanovectors coated with membranes from activated leukocytes enriched with inflammation-related receptors.

In the present study, native EVs from grapefruit juice have been characterized by their size, quantity, and morphology by commonly used in nanoparticle size and shape interpretation methods, including NTA, DLS and AFM. As the population of isolated EVs is usually heterogeneous in size, origin and molecular constituents, different techniques applied for their characterization have some limitations^31^. Cryo-EM method based on direct imaging of single particles in their close-to-native state provides the most reliable data for extracellular vesicle size and shape determination. Indeed, this technique allowed us to obtain high-quality images of grapefruit membrane-enclosed vesicles and accurately determine their size distribution. Most of the isolated GF-EVs were single, round-shaped, with sizes ranging from 30 to 55 nm. To our knowledge, this is the first visualization of plant-derived vesicles with a high enough resolution to allow the estimation of their lipid bilayer membrane thickness as 5.3 ± 0.8 nm. In general, cryo-EM data are in accordance with the analysis of GF-EVs by other methods based on the single or the ensemble particle sizing approaches. Previously, several studies have reported that ultracentrifugation may cause aggregation or morphological changes of EVs^32,33^, including double and multilayer structures, which could be observed in substantial amounts in many exosome samples, prepared from human biological fluids or cell culture medium^34–36^. While GF-EVs were purified with more stringent centrifugation, Cryo-EM visualization did not show any significant nanovesicle aggregation or double particle formation in the obtained suspension. The data encouraged us to hypothesize that native GF-EVs isolated by ultracentrifugation can be used as carriers of exogenous molecules. Thus, we loaded the GF-EVs with protein cargoes using sonication technique, that potentially may also cause the structural distortion of membranes, resulting in forming of the fused vesicles. However, we did not find significant changes in morphology and size distribution of the loaded by sonication GF-EVs.

Using in vitro models, we have demonstrated the significantly more efficient uptake of fluorescently labelled proteins HSP70-AF647 or BSA-AF647 loaded to GF-EVs by human cells compared to the free proteins. Since HSP70 is one of powerful anti-apoptotic molecules, we incubated DLD1 human cancer cells with GF-EVs loaded with the protein and found that this procedure caused enhanced cell resistance to etoposide suggesting that the chaperone retained its cytoprotective activity being embodied into vesicles. These results demonstrate that the native grapefruit vesicles are highly efficient carriers of functional exogenous protein into human cells in vitro.

Being one of the widely used labeling method for studying human exosomes *in vivo*^37^, membrane labelling carries the risk of exosome aggregation and ambiguities in results, since modification of surface proteins by the covalent binding of probes may change the properties of exosomes to interact with target cells or tissues^38^. Therefore, for in vivo analysis of vesicle distribution, we injected GF-EVs loaded with ^125^I-BSA intravenously to mice. The applied loading of GF-EVs with labelled protein does not modify the surface of the vesicles. In vivo analysis of the distribution of protein-loaded GF-EVs demonstrated their effective uptake by the majority of animal tissues. The biodistribution patterns for GF-EVs was typical for human exosomes in mice (reviewed in ^39^). The results of our study clearly indicate a high potential of native GF-EVs for the safe delivery of exogenous proteins into mammalian cells and tissues. In general, these results and the data published earlier^13,19,20,30^ provide a strong basis for further studies and development of plant vesicle delivery systems for their use in novel therapeutics and precision medicine.

## Materials and methods

### Reagents

The following reagents were used in the study: Alexa Fluor 647 Protein Labeling Kit (Invitrogen, USA), Bradford reagent (BioRad, USA), Clarity Western ECL Blotting Substrate (Bio-Rad, USA), mouse monoclonal antibody to human HSP70 (Abcam, ab2787). Recombinant human HSP70 protein was isolated and chaperone activity of the protein was established by the specially designed assays^40^. All other reagents used in the study were obtained from Sigma-Aldrich (USA).

### Isolation and purification of vesicles from fruit parts of Citrus x paradisi (grapefruits)

Grapefruits were purchased from a local market and washed three times with distilled water. The juice was extracted using a household citrus juicer. The collected volume (450 mL) of initial juice was sequentially centrifuged at 1200×*g* for 20 min, 3 times at 3000×*g* for 20 min, 10,000×*g* for 60 min, and 15,000×*g* for 60 min to remove large particles and cellular debris. The supernatant was centrifuged again at 10,000×*g* overnight. The sequential ultracentrifugation method was further applied; it included centrifugation of the final supernatant on a Beckman Optima L-90 K ultracentrifuge (Ti45 rotor, Beckman Coulter, USA), at 150,000*g* for 2 h. After first centrifugation, the supernatant was removed, and the pellet was carefully resuspended in 1 mL of phosphate-buffered saline (PBS) using gentle swaying overnight, then the volume was adjusted to 5 mL and re-centrifuged at 150,000*g* for 2 h (SW 55Ti rotor, Beckman Coulter, USA). The resulting pellet was re-suspended with gentle shaking in 500 μL of PBS for at least 1 h at 4 °C. Final samples of grapefruit-derived nanovesicles were aliquoted, rapidly frozen in liquid nitrogen and stored at − 80 °C until the analysis.

### Nanoparticle tracking analysis (NTA)

The size of GF-EVs and their concentration in suspensions were determined by NTA using the NanoSight LM10 (Malvern Instruments, UK) analyzer, equipped with a blue laser (45 mW at 488 nm) and a C11440-5B camera (Hamamatsu Photonics K.K., Japan). Recording and data analysis were performed using the NTA software 2.3. The following parameters were evaluated during the analysis of recordings monitored for 60 s: the average hydrodynamic diameter, the mode of distribution, the standard deviation, and the concentration of vesicles in the suspension.

### Dynamic light scattering (DLS)

The distribution of GF-EVs in size was evaluated by the method of DLS using a PLSS laser correlation spectrometer (INTOX MED LLC, Russia) as described earlier^41^. Measurements were carried out at 25 °C. For each sample, the particle size distribution curves were plotted according to the results of three measurements.

### Atomic force microscopy (AFM)

Detection of GF-EVs was carried out by AFM. Briefly, sample of GF-EV suspension in PBS was diluted 50-fold with deionized water, and 0.5 µL aliquots were deposited onto freshly cleaved mica. After drying completely at room temperature, the mica surface was flooded with the excess of deionized water to dissolve salt. The remaining water was removed with compressed air after a 5-min incubation. The sample topography measurements were performed in semi-contact mode using the atomic force microscope “NT-MDT-Smena B” with a NSG03 probe (NT-MDT, Russia). The images were analyzed using “Gwyddion” software^42^.

### Cryo-electron microscopy

Direct visualization of the grapefruit-derived vesicles was performed by Cryo-EM as described previously for vesicles isolated from human fluids^34^. The aqueous solution of the sample was applied on glow-discharged lacey carbon EM grid, which was then plunge-frozen into the precooled liquid ethane with Vitrobot Mark IV (ThermoFisher Scientific, USA). The samples were studied using a cryo-electron microscope Titan Krios 60–300 TEM/STEM (ThermoFisher Scientific, USA), equipped with TEM direct electron detector Falcon II (ThermoFisher Scientific, USA) and Cs image corrector (CEOS, Germany) at accelerating voltage of 300 kV. To minimize radiation damage during image acquisition low-dose mode in EPU software (ThermoFisher Scientific, USA) was used.

### Loading of grapefruit-derived vesicles with proteins

A combination of passive and active cargo loading was used. Recombinant human HSP70 protein or BSA at a final concentration of 0.1 mg/mL was mixed with suspension of GF-EVs at a final concentration of ~ 5 × 10^11^ particles/mL and incubated overnight at 4 °C. Then, the mixture was sonicated at a frequency of 35 kHz for 15 min by the Bandelin SONOREX SUPER ultrasonic bath (Bandelin Electronic GmbH & Co. KG) at room temperature, and incubated for additional 90 min at 4 °C. To remove the excess of free proteins, the vesicles were purified using ultrafiltration through a 100-kDa filter (Amicon, Millipore) and ten times washing with PBS. The first and tenth eluates in filtration procedure (F1 and F10) were used as controls in further experiments. The obtained suspension of protein-loaded grapefruit vesicles was adjusted to the starting volume of the initial suspension of GF-EVs with PBS. The final concentration of loaded GF-EVs was established by NTA. The total protein amount of GF-EVs, F1, F10, and GF-EVs loaded with HSP70 was determined using Bradford reagent (BioRad, USA). For some experiments Alexa Fluor 647 labeled variants of HSP70 and BSA (HSP70-AF647 and BSA-AF647) were used for loading to GF-EVs in a similar manner. Loading efficiency of GF-EVs with labeled proteins, as well as the efficiency of washing the vesicles from free protein, was analyzed by measuring the fluorescence of the samples with a spectrofluorometer (Hitachi F-7000).

### Western blotting

The presence of HSP70 protein in the samples of loaded GF-EVs was determined by western blotting. The purified samples of GF-EVs loaded with recombinant HSP70 were incubated at 4 °C for 30 min with 20 µL of lysis buffer (7 M urea, 2 M thiourea, 4% CHAPS, 5 mM PMSF, 1% DTT). The same number of vesicles isolated from grapefruit (without loading procedure) was analyzed in parallel. The protein samples were diluted in Laemmli buffer (BioRad, USA), subjected to 10% SDS-PAGE containing 0.1% SDS, and transferred to the PVDF membrane (Thermo Scientific) using the Trans-Blot Turbo Transfer System (BioRad, USA). Immunoblotting was performed according to the Blue Dry Western protocol^43^. Mouse monoclonal antibodies to HSP70 (Abcam, ab2787) were used as primary antibodies at 1:200 dilution. Horseradish peroxidase-conjugated goat antibodies against mouse immunoglobulins were used as secondary antibodies at 1:10,000 dilution. Chemiluminescent detection of the protein bands was performed with Clarity Western ECL Blotting Substrate (Bio-Rad, USA) and Thermo Scientific CL-XPosure Films (Thermo Fisher Scientific, USA). Recombinant human HSP70 protein (10 µg) was used as a positive immunodetection control.

### GF-EV-mediated delivery of mammalian proteins into human cells

HCT116 and DLD1 human colon cancer cell lines were obtained from the Cell Culture Collection, Institute of Cytology of the Russian Academy of Science. Cells were cultured in DMEM-F12 (BioLot, Russia) containing 10% FBS (Hyclone) at 37 °C, 5% CO_2_. Cultured DLD1 cells were stably transfected with pcDNA3 (DLD1-*scr*) or pcDNA3-HSP70 (DLD1-*hsp70*) plasmids as previously described^44^. The resulting cells expressed GFP alone or *hsp70* shRNA and GFP, respectively.

Mononuclear cells were freshly isolated from blood samples collected from healthy donor volunteers. This study has been approved by the NRC “Kurchatov Institute”-PNPI Ethics Committee (protocol No. 5, dated December 08, 2020) and conducted according to the principles expressed in the Declaration of Helsinki. All samples were collected after written informed consent. Peripheral blood mononuclear cells (PBMC) were separated from the buffy coat within 24 h after obtaining the blood specimens. The lymphocyte and monocyte band (PBMC layer) was isolated from healthy blood donor buffy coat by Ficoll-Paque density gradient centrifugation according to method described in ^45^.

For protein delivery assays, PBMC, HCT-116 or DLD1 cells were seeded on 12-well plates at a density of 10^5^ cells/well. In order to deliver the exogenous proteins, purified samples of protein-loaded GF-EVs were co-cultured with the recipient cells. The number of loaded vesicles was determined by NTA and the equivalent number of vesicles was added (10^6^ vesicles/cell). After 1 h of incubation, the cells transfected via GF-EVs with fluorescently labeled HSP70 or BSA were trypsinized and collected, then washed three times with PBS and analyzed by Confocal microscopy (LEICA TCS SP5X) and Flow cytometry (CytoFLEX, Beckman Coulter). The monocyte or lymphocyte populations were determined by flow cytometry based on forward and side scatter (FSC *vs*. SSC) gating (Supplementary Fig. S3) as previously described^46,47^.

To determine the functionality of the HSP70 protein delivered by GF-EVs, the proliferative activity of recipient cells in the presence of etoposide was analyzed. DLD1-*scr* and DLD1-*hsp70* cells were seeded in 16-well xCELLigence E-Plate at a density of 10^4^ cells/well and cultured overnight. Then grapefruit vesicles loaded with HSP70 and control vesicles (not loaded with HSP70) at concentration of 10^6^ particles per cell were added. Control samples, including only HSP70 protein (2 μg/mL), were incubated in parallel. After 4 h, etoposide at a concentration of 20 μM was added. A comparative analysis of the proliferative activity of cells in the presence of etoposide was performed in real time using instrument xCELLigence RTCA DP System (ACEA Biosciences, Inc, USA). All samples within each experiment were in duplicates.

### Loading of grapefruit-derived vesicles with ^125^I-labeled proteins

Radioactive iodine Na^125^I was purchased from Khlopin Radium Institute (St. Petersburg, Russia). Bovine serum albumin was radioactively labeled according to a modified procedure described in ^48^. The reaction mixture contained 15 μl of 50 mM KI, 10 μl of Na^125^I (0.8 GBq/ml), 20 μl of 0.5 M KH_2_P0_4_, 20 μl of 0.5 M Na_2_HP0_4_, and 200 μl of 10% BSA. Following the sequential mixing of reaction components, a 100 μl of chloramine T (20 mg/ml) was added to start labeling for exactly 40 s at room temperature. Then the reaction was stopped by addition of 200 μl of 0,1 M Na_2_S_2_O_5_. The labeled protein has been separated from free iodine by gel filtration chromatography on 3 ml column packed with Sephadex G10 (GE Healthcare). ^125^I-labeled BSA (^125^I-BSA) was used for loading into GF-EVs similar to the experiments described above. Loaded GF-EVs were purified from the free protein by ultrafiltration on Amicon 100 kDa cut-off concentration units. Loading efficiency of GF-EVs with ^125^I-BSA protein, as well as the efficiency of washing the vesicles from the free protein, was analyzed by measuring the radioactivity of the samples using gamma-vials in scintillation counter Tri-Carb 5110 (PerkinElmer, USA). The absence of free ^125^I in the final samples was controlled by thin layer chromatography (Supplementary Fig. S4).

### Ex vivo gamma counting of excised organs

The outbred CD-1 IGS mice from NRC “Kurchatov Institute”—Rappolovo breeding nursery (St. Peterburg, Russia) were used in this study. All experiments with laboratory animals were performed in compliance with the legislation of the Russian Federation and the rules of bioethics. All manipulations were conducted in compliance with the protocol approved by the Local Bioethics Commission of the NRC “Kurchatov Institute”—PNPI, Commission decision No. 9 dated December 08, 2020. The study was carried out in compliance with the ARRIVE guidelines^49^.

Female mice (24.8 ± 2.0 g, 6–8 weeks old) were injected intravenously with GF-EVs loaded with ^125^I-BSA (~ 250,000 CPM per mouse) or free ^125^I-BSA carrying equivalent amount of radioactivity. After 2 h, mice were sacrificed (n = 3 per group). Blood and major organs (brain, lungs, liver, spleen, kidneys, heart, bladder, uterus and ovaries) were collected, organs were washed by 0.1 M NaCl, weighed and placed in scintillation vials. Samples were counted in a Tri-Carb scintillation analyzer (PerkinElmer, USA) together with radioactive dose standards. Radioactivity readings (counts per minute—CPM) were expressed as percentage of injected dose per gram of tissue (%ID/g) or percentage of dose in a gram of blood per gram of tissue (%RBD/g). Data were expressed as the mean ± SD of sample triplicates.

### Statistical analysis

Statistical analysis was performed using SPSS 22.0. Experimental data are expressed as the mean ± the standard deviation (SD). The unpaired t-test or one-way ANOVA followed by multiple comparisons by Tukey’s post-hoc analysis was used to assess differences between groups. *P* < 0.05 was considered statistically significant. Statistical significance of the data is designated with asterisk(s): *, *p* < 0.05, **, *p* < 0.01, and ***, *p* < 0.001.

## Supplementary Information

Supplementary Video.

Supplementary Figures.
