# Supplementary material for: Delivery of functional exogenous proteins by plant-derived vesicles to human cells in vitro
Source: Sci Rep. 2021 Mar 22;11:6489. doi: 10.1038/s41598-021-85833-y (PMC7985202; doi:10.1038/s41598-021-85833-y)
Supplement: Supplementary file 2 — Supplementary Figures. [file 41598_2021_85833_MOESM2_ESM.pdf]

Supplementary Materials

Delivery of functional exogenous proteins by plant-derived vesicles to human cells in vitro

Luiza Garaeva, Roman Kamyshinsky, Yury Kil, Elena Varfolomeeva, Nikolai Verlov, Elena Komarova, Yuri Garmay, Sergey Landa, Vladimir Burdakov, Alexander Myasnikov, Ilya A. Vinnikov, Boris Margulis, Irina Guzhova, Alexander Kagansky, Andrey L. Konevega and Tatiana Shtam

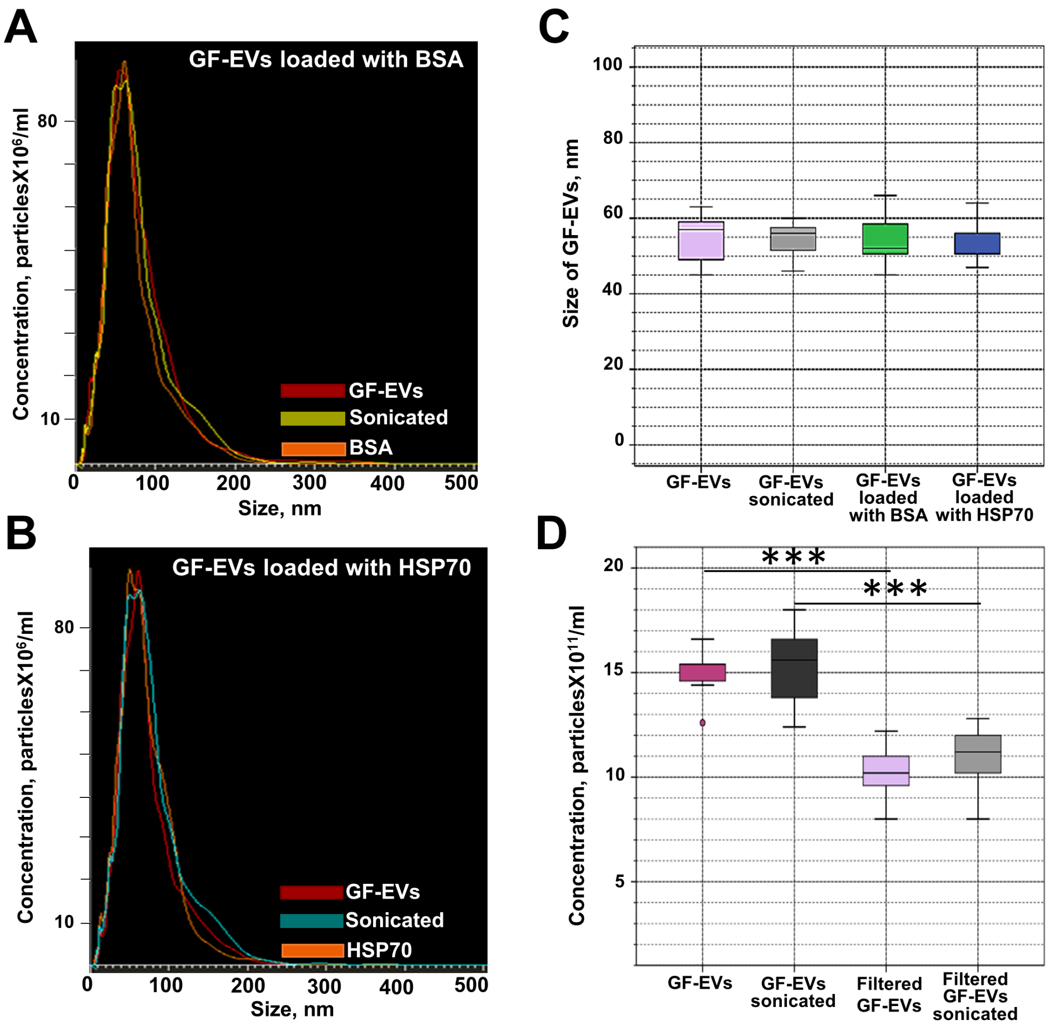

Supplementary Figure S1. Size and concentration distribution of grapefruit-derived extracellular vesicles (GF-EVs) loaded with exogenous proteins measured by NTA. (A) Nanoparticle tracking analysis (NTA) of size and concentration in the samples of GF-EVs, sonicated GF-EVs and GF-EVs loaded with BSA protein. **(B)** NTA of size and concentration in the samples of GF-EVs, sonicated GF-EVs and GF-EVs loaded with HSP70 protein. **(C)** The size comparison of GF-EVs, GF-EVs after sonication and GF-EVs loaded with BSA or HSP70 protein. No statistically significant differences were found between the groups (p=1 as determined by one-way ANOVA followed by Tukey's multiple comparisons post-hoc test). **(D)** Concentrations of GF-EVs, sonicated GF-EVs and the same samples purified using ultrafiltration through a 100-kDa filter. The concentration of filtered samples with GF-EVs or GF-EVs after sonication decreased significantly in comparison with the samples without filtration. ***, $p < 0.001$ as determined by one-way ANOVA followed by Tukey's multiple comparisons post-hoc test.

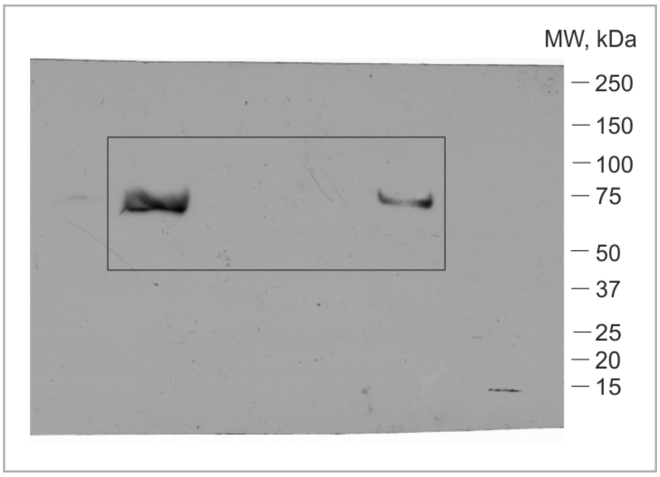

Supplementary Figure S2. Full-length Western blot image used to generate panel in **Figure 4D**.

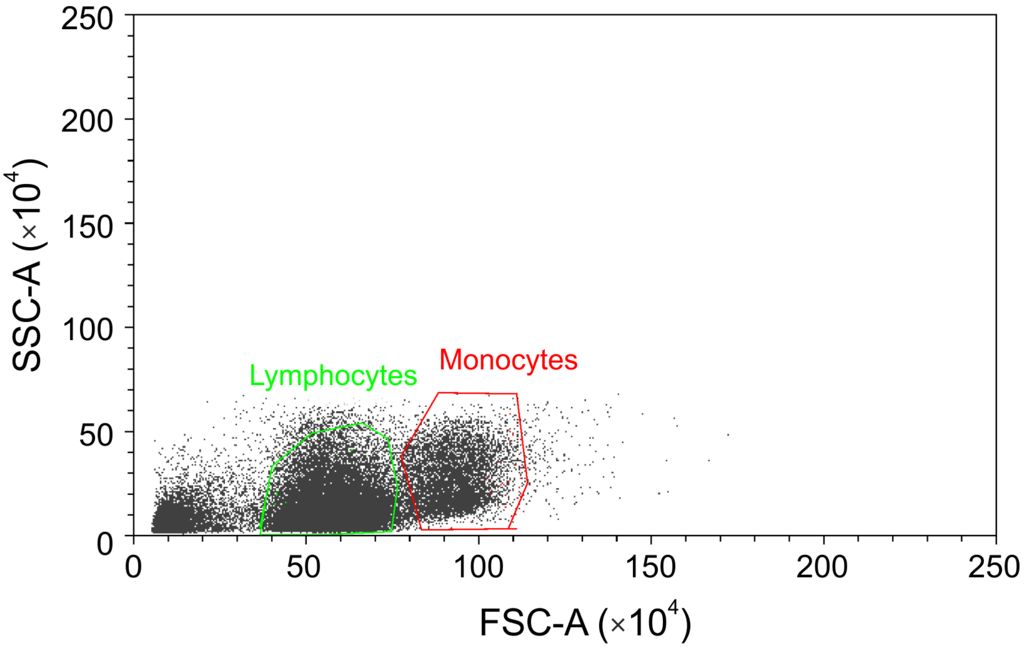

Supplementary Figure S3. Flow cytometric analysis of human peripheral blood mononuclear cell (PBMC) populations. Gating of monocytes (red plot) and lymphocytes (green plot) based on their forward and side scatter (FSC/SSC) properties.

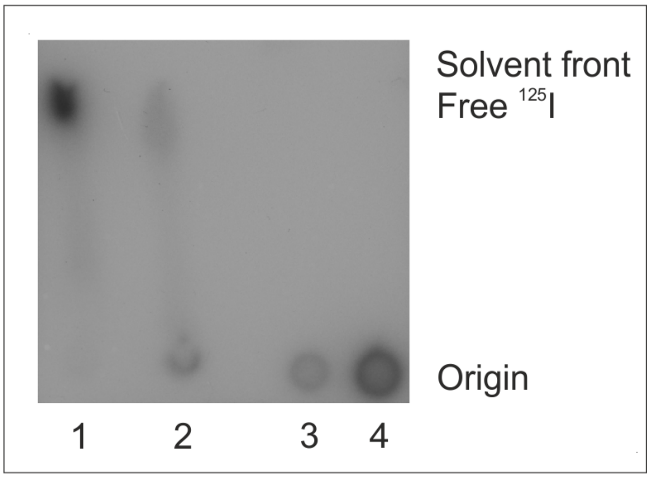

Supplementary Figure S4. Thin-layer chromatography (radiography). Lanes: 1, free ^{125}I; 2, ^{125}I-labelled BSA (^{125}I-BSA); 3, ^{125}I-BSA separated from free iodine by gel filtration chromatography; 4, grapefruit-derived extracellular vesicles (GF-EVs) loaded with ^{125}I-BSA.

Supplementary Video S1. Accumulation of fluorescent protein delivered to the recipient cells by grapefruit-derived extracellular vesicles (GF-EVs).
